# The several faces of the medicalization of birth. Italy and its peculiarities

**DOI:** 10.3389/fsoc.2023.1000518

**Published:** 2023-05-30

**Authors:** Elena Spina

**Affiliations:** Dipartimento di Scienze Economiche e Sociali, Università Politecnica delle Marche, Ancona, Italy

**Keywords:** medicalization of pregnancy and birth, maternity model of care, European comparison, regional differences in Italy, childbirth policies

## Abstract

**Background:**

Medical-scientific advances in maternal care gradually improved the health of mothers and new-borns. However, this has contributed to increasing levels of medicalization, defined as the overuse of medical interventions even in low-risk pregnancies and childbirths. In Italy pregnancy and birth still appear to be rather medicalized than in the rest of Europe. Moreover, the uneven distribution of these practice over the territory appears to be evident. The purpose of this article is to both highlight and explain the Italian peculiarity in terms of high medicalization of childbirth and its territorial variability.

**Theoretical framework:**

The extensive literature on medicalization of childbirth was systematized by some scholars who use childbirth as a case study to distinguish four meanings of medicalization, by classifying them into two generations of theories. Alongside this literature several studies attempted to interpret differences in maternity model of care showing the important role played by path dependence.

**Results:**

In the European scenario, Italy stands out for its high percentage of cesarean sections, but also for its excessive recourse to antenatal visits during pregnancy and the application of interventions during labor and vaginal births. Going into regional detail, however, Italian situation appears rather uneven: relevant differences emerge in relation to medicalization of both pregnancy and birth.

**Discussion:**

The article explores the possibility that areas whit different sociocultural, economic, political and institutional background may have introjected different meanings of medicalization, thus reproducing different maternity models of care. In fact, the simultaneous presence, in Italy, of four different meanings of medicalization seems to be rooted. Even with some similar traits, different conditions and situations emerge in different geographical areas, leading to the prevalence of one meaning rather than another and resulting on different outcomes in terms of medicalization.

**Conclusion:**

The data presented in this article seem to deny the existence of a national maternity model of care and. On the contrary, they confirm the idea that medicalization is not necessarily linked to the different health conditions of mothers in different geographical areas and that a path dependent variable is able to explain it.

## 1. Background

Medical-scientific advances in maternal care gradually improved the health of mothers and new-borns. However, this has contributed to increasing levels of medicalization, defined here as the overuse of medical interventions even in low-risk pregnancies and childbirths.

In many European countries, attempts have been made to counter this trend by promoting practices and reorganizing pregnancy and childbirth pathways to demedicalise them (Ten Hoope-Bender, 1997; Dalton, 2009; World Health Organization, 2009; Smith et al., 2012; Crenshaw, 2014; Donati, 2021).

In Italy this experience still appears to be rather medicalized: the most striking example is the high percentage of cesarean sections (CS), among the highest in Europe (OECD, 2021), although slightly decreasing in recent years (Ministry of Health, 2021). Surgical childbirth, however, is only the most obvious demonstration of medicalization, which also takes place during pregnancy (with excessive recourse to check-ups and ultrasound screening) and during spontaneous births (through the application of procedures such as induced rupture of membranes, episiotomy, use of oxytocin, etc.).

Moreover, another peculiar aspect of the Italian case concerns the uneven distribution of these practices over the territory, which makes it difficult to identify the existence of a national maternity model of care.

In this regard, what should be pointed out is that maternity care appears to be similar to the broader healthcare sector, showing the same characteristics in terms of distributive justice and, in particular, in terms of equity of resources, thus limiting equity in access and equality in outcome (Vicarelli and Spina, 2020). Since before the establishment of the Italian National Health Service (INHS) in 1978, Italy showed strong health inequalities. By centralizing and universalising care, the INHS tried to reduce them, with unsatisfactory results and wide inequalities remained. They worsened during'80, as a result of the driving forces toward regionalism which intensifying in the first 2000, due to the constitutional Law no. 3 2001 which expanded the role and competences of regional autonomies. Starting from 2008, in fact, in the face of the requests for differentiated autonomy by some central and northern Regions, an attitude of devaluation of the less performing areas seems to have set in. This trend can be defined in terms of “reduced or sufficient universalism,” which is moreover identified as one of the four distortions characterizing the INHS today (Giarelli and Vicarelli, 2021) together with the distributive one, relating to the profound social inequalities of health, the cultural one linked to the orientation toward illness and the consequent medicalization of health, and the functional one, which can be summed up in the concept of hospital-centrism, that is the still persistent centrality of the hospital as the pivot and backbone of the healthcare system. The political and organizational choices taken to manage the regional health system led regional health services to function in different ways and to achieve different degrees of performance. In the light of these peculiarities, several scholars hypothesized the existence of sub-regional health models of care (Neri, 2006, 2008; Pavolini, 2008; Bertin, 2013; Servetti, 2018), based on different functioning mechanisms. Almost all of them trace, albeit in different ways, the geographical tripartition north-center-south.

The aim of this paper is to show the need for examining some contextual variables (cultural, institutional, political, professional) to understand the reasons for the configuration assumed by maternity care in different places in which it is provided. Just as we have attempted to do in the case of welfare or health systems, by searching for sub-national models, the inequalities found on the levels of medicalization of birth in Italy would require an in-depth examination looking for territorial peculiarities.

The article opens with a review of the literature on medicalization of birth as well as on the attempts to modelized maternity care (first paragraph). Using international (OECD, Eurostat, Euro-Peristat, WHO) and national data (Ministry of Health, ISTAT, Save the Children, etc.) it presents the Italian case in the European scenario, then going down to a sub-national level to bring to light the lack of homogeneity that characterizes the peninsula (second paragraph). An attempt to explain different levels of medicalization in Italy using theoretical framework set out in the first part of the paper is presented in the third paragraph. Some reflections on the future of maternity care in Italy is offered in the conclusions.

## 2. Theoretical framework

There is an extensive literature focusing on the medicalization of birth and childbirth. An interesting attempt to systematize it was made by Christiaens and van Teijlingen (2009) who use childbirth as a case study to distinguish four meanings of medicalization by classifying them into two generations of theories.

The first generation emphasizes the hegemony of the medical profession and is based on a relatively simple health care system with health professionals and patients as the protagonists. It includes three different meanings.

The first, which can be traced back to the studies of Foucault (1963) and Freidson (1970), refers to the stimuli offered by Parsons (1958). Here, medicalization coincides with the professionalization of medicine and the development of medical knowledge: these phenomena have enabled professionals to gain increasing control over the female body as a precondition for social order. The medicalization of birth is expressed in the mechanical view of the maternal body, regulated according to its reproductive capacity. This legitimizes the use of technology as well as the change of setting (home vs. hospital) and the replacement of midwifery care with medical one. As pregnancy and childbirth are institutionalized, due to the growing conviction that specialized care was necessary to give birth to healthy children, children, and no longer the mothers, become the protagonists of this event.

The second meaning, which refers to the work of Zola (1972) and Conrad (1992), sees medicalization as the increasing medical control over more domains of daily life (Branckaerts, 1982). Zola (1972), in particular, identifies the causes of the increased authority of medicine in the extension of its jurisdiction, the discovery of new pathologies and the creation of new dysfunctions: in other words, he states, health is an important value in society and the medical profession exerts control over what should or should not be done.

Conrad (1992) considers medicalization as “a process by which non-medical problems become defined and treated as medical problems, usually in terms of illnesses or disorders” (p. 209). He argues that medicalization can occur at the conceptual, institutional and/or interactional level and that users themselves can contribute to it, especially when they cannot rely on alternative social responses to those provided by medicine and its representatives. Referring to this second meaning, the medicalization of birth hinges on the concept of medical imperialism. Research in this field, by strongly criticizing the way professionals control the birth process and women's bodies, emphasizes that the use of medical technology results in alienation through the erosion of women's control over the birth process (Davis-Floyd, 1987, 1994; Martin, 2001). This position, however, clashes with the increasing satisfaction shown by women, who feel comfortable in the hospital environment under medical supervision: many women desire and actively seek medical control over the unpredictable process of birth (Sargent and Stark, 1989; Lazarus, 1994). This paradox leads one to question that the medicalization of childbirth is the result of medical imperialism and to consider the active role of women in fostering this trend.

The third meaning of medicalization goes back to the studies of Illich (1976), who first argued that the rise of medicine, considered as a powerful institution, restricts the individual capabilities that are necessary for personal growth and self-care. Illich defines the adverse consequences of medicine with the concept iatrogenesis (Branckaerts, 1982) by identifying three forms: clinical, social and structural. These forms are all present in the childbirth and midwifery literature. The clinical iatrogenesis is implicit in studies concerning the adverse impact of medical procedures on women's experiences (Ryding et al., 1998, 2000; Munro et al., 2005), psychological wellbeing (Fisher et al., 1997) and mother-child interaction (Rowe-Murray and Fisher, 2001). These studies also inform about structural iatrogenesis, as often the loss of control is predicted outcome of interventionist births. As a result, the medical model is criticized for undermining women's autonomy in childbirth (Cahill, 2001) and is opposed by the midwifery one, which, on the contrary, stimulate self-confidence and empowerment (Thachuk, 2007). Finally, social iatrogenesis reflecting unanticipated consequences of the sick role, is illustrated by studies about the conflict women experience between motherhood and employment (Johnston and Swanson, 2006; Grice et al., 2007) and impact of the transition to parenthood on personal wellbeing and marital satisfaction (Keeton et al., 2008; Lawrence et al., 2008; Moller et al., 2008).

Social, political and cultural transformations that took place in industrialized countries from the 1980s onwards favored a new social reality and the transition to the second generation of theories. These processes of change contribute to the decline of medical authority, witnessed by studies introducing the concepts deprofessionalization and proletarianization (Haug, 1973) as well as by those focusing on the growing influence of pharmaceutical companies (Conrad and Leiter, 2004; Arney and Rafalovich, 2007; Rose, 2007), insurance markets, on commodification of health care and on the technological revolution. In this context the roles of each player in the field of health care have changed: that of physicians become increasingly complex (Metzl and Herzig, 2007); patients, more and more proactive, become consumers (Hartley, 2002). A growing number of studies emphasizes the active role of mothers and fathers in the doctor-patient interaction and in decision-making regarding pregnancy and birth (VandeVusse, 1999; Shorten et al., 2005; Van der Hulst et al., 2007): in the idea that interaction with the professional is very complex, Zadoroznyj (2001) argues that pregnant women taking up the roles of both consumers and “patients” so they can be considered as reflexive consumers. A new medicalization idea emerges (the fourth) based on the optimalization of normal characteristics. The idea is that medicine, by shifting its focus from the population to the subject, in an increasingly individualistic and personalized perspective, moves from a logic of control to a logic of transformation. Biomedicalization thus acts on the transformation of bodies in order to improve their capabilities (Clarke et al., 2009) thanks to technological advances that aim at optimizing health and not maintaining it.

In the childbirth field, this theme has been explored earlier. The medical model implicitly assumes that the imperfect female body can be ameliorated by medical interventions (Lane, 1995). The physiological event is optimized through medical control and technologies, that are developed to treat the disease and then are applied to “normal” behavior, thus creating a new consumer market. “The tendency to optimize normal characteristics should be understood in the light of the changing status of embodiment. The body is self-evidently present as a necessary condition enabling social action. The taken-for-grantedness of the body is broken down by the occurrence of pain, illness, discomfort (Leder, 1990) or pregnancy (Davidson, 2001). Today bodies are projects, which are managed, controlled and maintained as an inherent part of identities (Shilling, 1993). The development of a healthy, flexible and efficient body is decisive for the acquisition of social success and is compatible with the consumer culture (Shilling, 2002; Christiaens and van Teijlingen, 2009, p. 11).”

Within this new scenario, medicalization of birth is seen in terms of optimizing the “normal” conditions. In fact, it takes place in a changed context compared to the past and with increasing complexity where the asymmetry of positions between professionals and patients is partially reduced, thanks to the acculturation of the latter and the entry of new players in the medical market. As early as 1972, Zola argued: “The medicalizing of society is as much a result of medicine's potential as it is of society's wish for medicine to use that potential” (Zola, 1972, p. 182), indicating that medicalization is not a one-way street, but the result of intertwined social forces, both bottom up and top down.

Alongside this literature, several studies have attempted to interpret differences in maternity care domain. A useful analysis is that carried out by Benoit et al. (2005). By using a “decentred method,” the authors compare four countries (UK, Finland, the Netherlands and Canada) sharing several features, including political and economic systems, publicly-funded universal healthcare and favorable health outcomes, even belonging to substantially different welfare regimes. The analysis focuses on three key dimensions: 1. welfare state approaches to legalizing midwifery and negotiating the role of midwife in the division of labor; 2. professional boundaries in the maternity care domain; 3. consumer mobilization around maternity issues. They conclude shedding light on how different “welfare state organized their respective maternity care service, how boundary disputes in the health division of labor were contested, negotiated and renegotiated (or not), when and where consumer groups mobilized around maternity care issues and variation in the gendered dynamics across all three levels of analysis” (Benoit et al., 2005, p. 733).

A more recent study supports the idea that the configuration of maternity services is the result of the prevalence of different logics at different levels, of negotiations among professional groups that compete for an exclusive jurisdiction and of changing interests of lobbying groups that arise around pregnancy and childbirth (Kennedy and Kodate, 2015). More recently these same authors (Kennedy and Kodate, 2019) propose a comparative analysis of maternal services and policies prevailing in a larger number of countries, including Italy, looking at the influence exerted by contexts in the configuration of services. Here the role of path dependency in tracing national trajectories is highlighted.

All these studies show the important role played by the context in affecting maternity care domain. A limitation, however, can be seen in the lack of attention played in micro institutional context: focusing on national situation and its aggregated data, they underestimated the wide differences among local areas. As it will be seen for the Italian case, very strong geographical differences within each country, linked to different histories and subcultures, emerge. After all similar conclusions have been shown by studies that focus on inequalities in the provision of welfare and health services, thus assuming the existence (and simultaneous presence) of sub-models or sub-national variants (Neri, 2006; Servetti, 2018; Quaglia et al., 2021).

## 3. Results

As in the rest of Europe, considered a part of the world where giving birth is a safe practice, good perinatal outcomes have been achieved in Italy: significant reductions in the maternal mortality rate (2 per 100,000 live births in 2017, among the lowest in Europe where the average is 6) and infant mortality rate (in 2019 it was 2.4 per 1,000 live births against a European average of 3.4) (World Health Organization, 2019) have been registered. Several studies, however, show that the improvement in perinatal outcomes does not explain the medicalization of pregnancy and birth that remains still high and sometimes inappropriate (World Health Organization, 1985; Wagner, 2001).

The Italian data on medicalization of pregnancy and childbirth take on a different meaning if we look more closely at the different Italian regions: significant differences emerge between the north and south of the country.

Differences are observed in relation to perinatal outcomes (Table 1).

**Table 1:** Some demographic indicators.

**Region**	**Year 2020**		**Year 2018**				
	**Birth rate**	**Total fertility rate**	**Infant mortality rate**	**Neonatal mortality rate**			**Infant mortality rate**
				** < 1 day**	**1–6 days**	**1–29 days**	**1 month and over**
Piedmont	6.3	1.24	19.61	4.47	3.10	6.88	8.26
Aosta Valley	6.2	1.23	22.12	11.06	11.06	11.06	0.00
Lombardy	6.9	1.26	25.63	4.62	5.68	11.49	9.51
Trentino Alto Adige	8.5	1.52	33.21	6.23	13.49	17.64	9.34
Bolzano	9.6	1.69	32.17	9.46	11.36	13.25	9.46
Trento	7.4	1.35	34.46	2.30	16.08	22.97	9.19
Veneto	6.7	1.28	21.19	5.09	3.67	9.32	6.78
Friuli V.G.	6.2	1.26	21.71	5.11	3.83	7.66	8.94
Liguria	5.7	1.22	25.43	2.21	6.63	12.16	11.06
Emilia Romagna	6.7	1.27	23.15	2.47	5.25	13.89	6.79
Tuscany	6.1	1.17	21.72	5.23	5.23	8.45	8.04
Umbria	6.0	1.15	31.08	5.18	1.73	12.09	13.81
Marche	6.3	1.19	16.71	1.97	4.92	7.87	6.88
Lazio	6.6	1.18	30.37	9.02	9.96	15.66	5.69
Abruzzo	6.4	1.16	30.21	10.07	6.71	14.55	5.59
Molise	5.7	1.05	21.11	15.83	5.28	5.28	0.00
Campania	7.9	1.28	38.49	8.11	10.40	21.01	9.36
Apulia	6.7	1.17	32.85	6.92	7.26	17.98	7.95
Basilicata	6.3	1.12	40.36	2.69	13.45	21.52	16.14
Calabria	7.4	1.24	39.53	9.88	9.22	23.06	6.59
Sicily	7.7	1.32	40.35	8.36	11.56	20.91	11.07
Sardinia	5.1	0.95	25.43	10.60	5.30	7.42	7.42
**Italy**	**6.8**	**1.24**	**28.79**	**6.23**	**7.16**	**14.19**	**8.37**

Source: Ministry of Health (2021). Bold value relate to the Italian average.

Since infant mortality is negatively correlated with health, environmental and social conditions and with differing accessibility to health services, it can be an indicator of both differing levels of wellbeing between residents in different geographical areas and of unequal organization and supply of health services having dissimilar performance.

### 3.1. Medicalization of pregnancy

Italian data, although not recent, show how the percentage of complicated pregnancies^1^ by serious disorders has increased over time from 22.4% in 2005 to 25.4% in 2013 (ISTAT, 2017). The reason may lie in the higher incidence of pregnancies at an older age: the average age of mothers having their first child in 2020 is, in fact, 31.4 years, showing an increase of 2 years in the last decade and higher than that recorded at European level (29.5 years) (EUROSTAT data, 2020). In 2020, 61.4% of Italians who gave birth were between 30 and 39 years old; the percentage of women giving birth before the age of 20 was low (0.9%) and, by contrast, the share of women over 40 was significant (10.21%) (Ministry of Health, 2021). This makes Italy the country with the highest average age at childbirth despite having a low average number of children per woman.

An indicator used to value access to prenatal care, both internationally (Peristat indicators) and nationally (indicators of the Addendum to the LEA Grid^2^), is the timing of the first visit: the WHO recommends the first visit received before 12 weeks of pregnancy (World Health Organization, 2016) while the Italian recommendations reduce it to 10.

Against a high variability recorded at European level (Topcu et al., 2022) in Italy, ISTAT (2017) data, stopped in 2013, show how, if the percentage of women accessing for the first time within 3 months remains stable at 94%, there is a gradual tendency to anticipate this step to the first month of pregnancy (34.1% in 2013 against 28% in 2005) especially by women at their first experience, ahead of age, resident in the center-north and with a high level of education.

Moreover, a lot of antenatal visit and ultrasound scans are performed. According to the data from the Certificate of Birth Attendance (Ministry of Health, 2021) for the year 2020, 89.4 % of women underwent more than four antenatal visits (four are those recommended and offered by the National Health Service), and 74% underwent more than three ultrasound check-ups (36.1% underwent more than seven). If the risk of the pregnancy does not seem to have influenced the number of visits and ultrasound scans (Tables 2, 3), the professional who took charge of pregnancy may have conditioned the level of medicalization, as literature stated (ISTAT, 2017).

**Table 2:** Distribution of antenatal visits by course of pregnancy—Year 2020.

**Antenatal visits**	**Course of pregnancy**		**Total births**
	**Physiological**	**Pathological**	
None	0.7	1.1	0.8
≤ 4	9.9	8.9	9.7
More than 4	89.4	89.9	89.5
Total	100.0	100.0	100.0

Source: Ministry of Health (2021).

**Table 3:** Distribution of ultrasound scans by course of pregnancy—Year 2020.

**Course of pregnancy**	**Ultrasound scans**		
	**2018**	**2019**	**2020**
Physiological	5.61	5.66	5.66
Pathological	5.57	5.65	5.55
Not specified	4.16	4.93	5.13
Total	4.94	5.62	5.60

Fonte: Ministry of Health (2021).

It is thus clear that the medicalization of pregnancy is not associated with the risk taken, but with other factors.

Important differences are observed at geographical level. Southern women undergo antenatal examinations (76.1%) more than those in the center and north. These data reveal inequalities on the side of public supply, considering how 68% of southern women in poor economic conditions nevertheless rely on a private professional, against a percentage of 41.2% among central-northern women in the same conditions (ISTAT, 2017). Regarding ultrasound scans, data (Ministry of Health, 2021) show that in the northern regions the average number fluctuates between 3.9 in the Autonomous Province of Trento and 5.9 in Liguria; in the central regions, compared to a value of 6.2 in Umbria, Lazio and Tuscany, it stands at 5.1, rising to 5.5 in Marche. In the south and the islands, on the other hand, the average number of ultrasound scans rises: in no region is it < 6, with a peak in Sardinia (7.4). Moreover, looking at women who have undergone 7 or more ultrasound scans, the differences with the regions of the center-north intensify considerably: in Sardinia 73.8% women have undergone more than 7 ultrasound scans, in Campania 66%, in Basilicata and Calabria 63.6 and 61.3%, respectively.

Relying, during pregnancy, on one professional rather than another may affect the level of medicalization (Bourgeault et al., 2001; Wagner, 2001; Benoit et al., 2005), as the Italian data seem to demonstrate. Women who rely on private gynecologists (66%), including those who also practice in public hospitals,^3^ mostly undergo more check-ups (ISTAT, 2017). Few women entrust their pregnancy care to midwives and overall, public community facilities (family counseling centers^4^) are underused.

Going down to regional level, the percentage of women who rely on a private gynecologist rises to 76.1% in the south compared to 57.9% in the center-north. Few, on the other hand, are the women who entrust their pregnancy care to a midwife: in the center-north, the percentages are on average higher than in the southern and island regions but are still very low.

The participation or non-participation in birth preparation courses can also impact on the level of medicalization of the birth process: Italian women who attend them seem to face labor and childbirth more consciously, serenely and collaboratively; at equal age, they are less frequently subjected to CS and other medicalized practices (trichotomy, enema) and more able to “negotiate an active role in decision-making processes” (Baglio et al., 2000, p. 475). Unfortunately, no up-to-date data are available, but an old survey conducted in Italy in 2000 showed that among women experiencing pregnancy for the first time, slightly more than half (53.8%) had attended an antenatal course (Baglio et al., 2000).

In this regard, the geographical gradient appears significant: in 2013 66% of women in the center-north have attended these courses; in the south the percentage is halved (33%). The lower participation in the courses in southern and insular Italy can probably be attributed to the lower offer of services as well as to the lack of knowledge, on the part of pregnant women, of the existence of the courses themselves (ISTAT, 2017).

### 3.2. Medicalization of birth

In addition to medicalization of pregnancy, birth also reproduces this trend in Italy. The medicalization of birth occurs not only through excessive recourse to CS, but also through medical and technological intervention as well as the use of inappropriate practices during labor and vaginal delivery.

CS is the most extreme form of medicalization and no evidence is available to support the association between the increasing use of CS and a reduction in maternal fetal risk or improvements in perinatal outcomes. In 2020, the CS rate is 31.4% (more frequent in women with Italian citizenship, 32.4%, than in foreigners, 27.2%), a percentage that, going well beyond the 10–15% range recommended by the World Health Organization (1985), places Italy at the top in Europe after Cyprus (54.8%), Romania (44.1%), Bulgaria (43.1%), Poland (39.3%) and Hungary (37.3%) (OECD, 2021).

**Figure 1:**
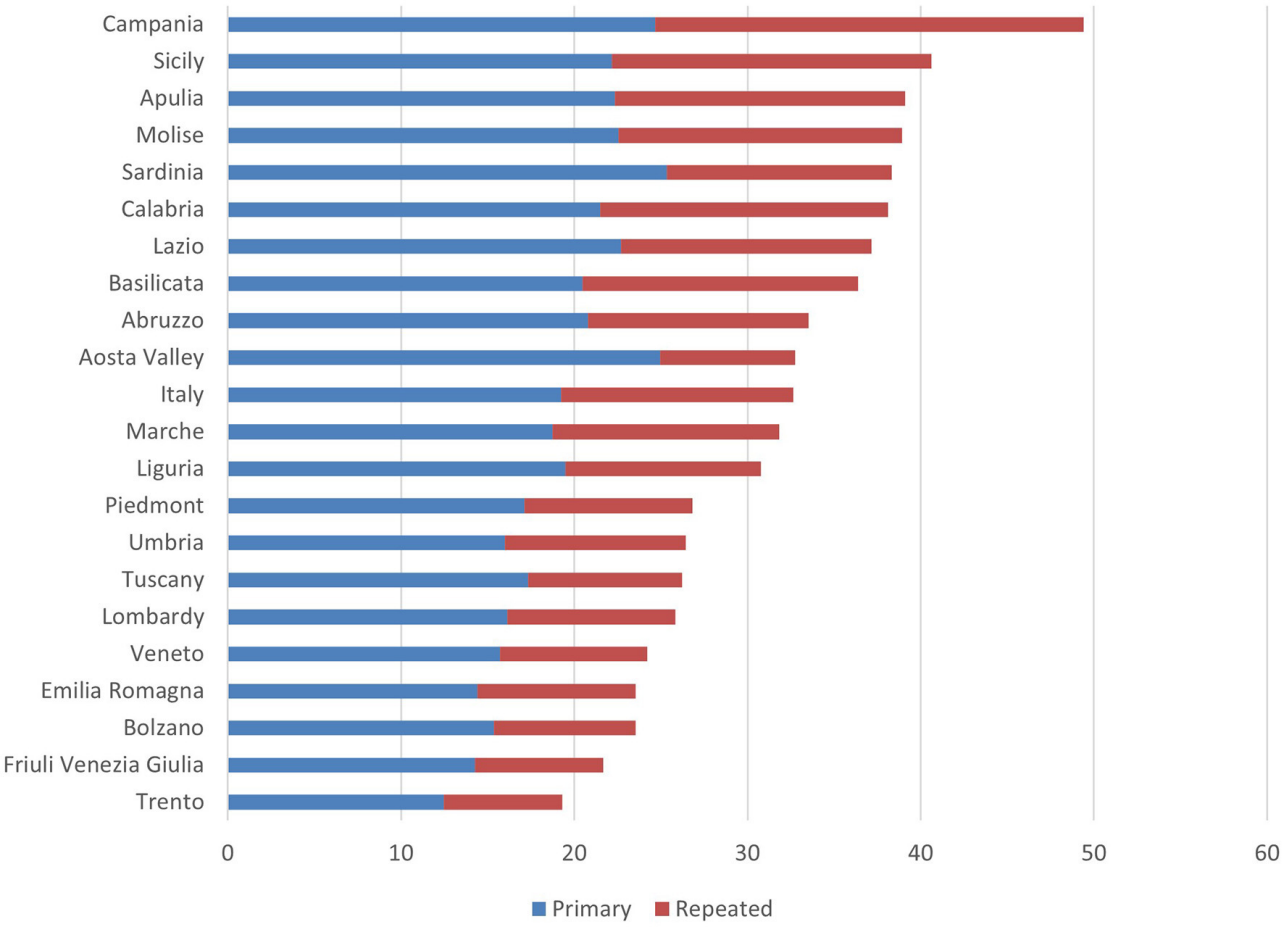
Proportion of deliveries with primary, repeat and total CS in Italy by region (values per 100)—Year 2020. Source: Osservatorio Nazionale sulla Salute nelle Regioni Italiane (2021).

Going down from the north of Italy to the south, for example, recourse to CS increases significantly (Figure 1). The Autonomous Province of Trento shows the lowest percentage (19.4%), but in the central north, except for Latium (35.9%) and Liguria (30.4%), rates never reach 27%. Campania holds the negative record with 50% of surgical births, followed by Sicily and Apulia (39.9 and 38.7%, respectively). The geographical gradient can be partly explained by the size and type of hospitals that appear to be relevant dimensions in its use: in the South, where there are many private clinics, mostly small, the CS rate is higher.

According to data from the Ministry of Health (2021), even in Italy, a high propensity for surgical practice is observed in private as well as in accredited hospitals (65.75 and 45.2%, respectively) compared to a percentage of 29.5% recorded in public hospitals (Table 4).

**Table 4:** Proportion of CS according to type and size of maternity wards—Year 2020.

**Class of births**	**Proportion of caesarean sections**			
	**Public**	**Private accredited**	**Private not accredited**	**Total**
0–499	34.12		65.75	33.68
500–799	30.19	50.62		32.16
800–999	28.88	48.89		32.22
1,000–2,499	28.76	45.58		30.94
2,500+	29.03	34.48		29.61
Total	29.50	45.27	65.75	31.30

Source: Ministry of Health (2021).

Moreover, hospitals with fewer than 800 deliveries per year, the incidence of CS is significantly higher than that observed on average at an overall level. The phenomenon is also correlated with the higher concentration of private facilities in the smallest birth classes (Ministry of Health, 2021).

The regional variability would therefore be due, first and foremost, to clinical and organizational factors, and, once again, to the unequal supply, rather than to differences in the population's state of health (Table 5).

**Table 5:** Distribution of CS according to the type of hospital facility—Year 2020.

**Region**	**Pubblic**	**Private**		**Total**
		**Accredited**	**Not accredited**	
Piedmont	26.7			26.7
Aosta Valley	21.7			21.7
Lombardy	23.0	24.7		23.2
Prov. Auton. Bolzano	23.4			23.4
Prov. Auton. Trento	19.6			19.6
Veneto	24.7			24.6
Friuli Venezia Giulia	20.5	20.3		20.5
Liguria	30.5			30.5
Emilia Romagna	23.6			23.6
Tuscany	20.5		80.0	20.5
Umbria	22.8			22.8
Marche	26.4			26.4
Lazio	34.6	39.2	65.5	35.9
Abruzzo	31.4			31.4
Molise	37.1			37.1
Campania	44.9	56.2		50.0
Apulia	38.3	43.5		38.7
Basilicata	35.3			35.3
Calabria	36.7	38.3		36.8
Sicily	38.2	51.3		39.9
Sardinia	35.9			35.9
**Total**	**29.5**	**45.3**	**65.8**	**31.3**

Fonte: Ministry of Health (2021).

Currently the trend in the use of CS appears to be decreasing, thanks mostly to the decline in primary (19.24%) vs. repeated (13.40%) CSs, which have fallen by 16.8% since 2011 (Osservatorio Nazionale sulla Salute nelle Regioni Italiane, 2021).

**Figure 2:**
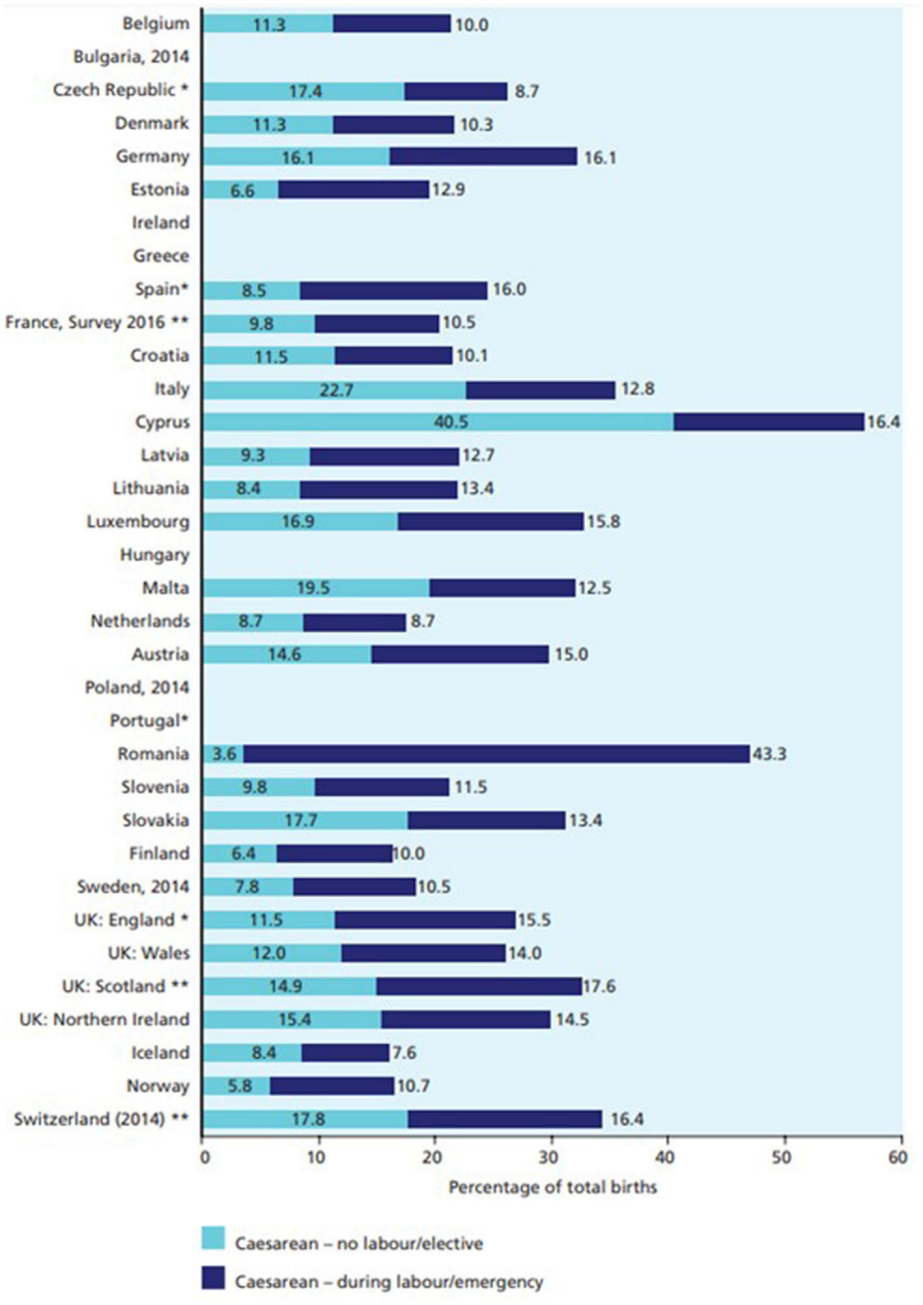
Proportion of births by type of caesarean in Europe—Year 2015. Source: EURO-PERISTAT Project (2018).

Most recent data suggest that previous cesarean is the most frequent cause of recourse to this practice: 96.5% of women who experienced a cesarean in a previous pregnancy in Italy repeated the same type of delivery although there is no evidence to support this practice. Looking at the type of CS, Italy also ranks first in Europe with reference to the percentage of planned cesarean (24.9%, compared to 12.9% of urgent ones), followed at a distance by Northern Ireland (15.5%), Malta (15.2%) and Germany (14.3) (Wise, 2015). Figure 2 shows the percentage of cesareans divided between planned and emergency for the year 2015. Rates for the former range from 3.6 to 40.5%, with a median of 11.3%; those for emergency cesareans range from 8.7 to 43.3% with a median of 12.9% (EURO-PERISTAT Project, 2018).

Differences among Italian regions can also be observed looking at the type of CS: according to ISTAT (2014), data in 2013 the 62.2% of CS were planned (this occurred more frequently in southern regions 64.6%), compared to 37.4% of emergency cesarean.

Looking at the implementation of vaginal birth after cesarean (VBAC), while it is true that since the late 1980s has significantly reduced the incidence of CS, the emergence of adverse events related to this practice has created an aversion among clinicians, causing a decrease in the number of vaginal births after a cesarean. On the other hand, no correlation emerges between the course of pregnancy and the use of this practice (Colais et al., 2018).

Italian data show 11.2% of VBAC in 2020; this phenomenon occurs more frequently in public birthplaces (12.8%) than in private hospitals (4.4%). Still uneven throughout the peninsula is the distribution of vaginal deliveries after a previous CS (Table 6). The prevalence given to this practice in public facilities anticipates the reasons for the geographical gradient. In the central north, in fact, the percentages are on average higher than those recorded in the south and on the islands: if in the Autonomous Province of Bolzano 39.4% of women who have undergone a previous CS give birth naturally, Campania has the negative record with a percentage of 2.8%. More generally in the South, except for Abruzzo (13.5), values never reach 10 percentage points.

**Table 6:** Regional distribution of vaginal deliveries after a previous CS by type of hospital facility—Year 2020.

**Region**	**Vaginal deliveries after a previous caesarean section**			
	**Public**	**Private**		**Total**
		**Accredited**	**Not accredited**	
Piedmont	21.5			21.5
Aosta Valley	11.9			11.9
Lombardy	18.7	23.9		19.3
Prov. Auton. Bolzano	39.4			39.4
Prov. Auton. Trento	31.9			31.9
Veneto	20.2	17.9		20.2
Friuli Venezia Giulia	28.1	28.9		28.1
Liguria	13.7			13.7
Emilia Romagna	20.1			20.1
Tuscany	14.7			14.7
Umbria	17.5			17.5
Marche	10.1			10.1
Lazio	8.4	2.8	12.7	5.9
Abruzzo	13.5			13.5
Molise	3.3			3.3
Campania	3.6	1.9		2.8
Apulia	4.3	3.6		4.2
Basilicata	3.1			3.1
Calabria	5.8	12.8		6.4
Sicily	5.7	1.3		5.1
Sardinia	9.9			9.9
Total	12.8	4.4	12.7	11.2

Source: Ministry of Health (2021).

If, therefore, the recourse to CS still appears to be quite high, even looking at vaginal delivery the level of interventionism, including forceps and vacuum, episiotomy,^5^ amniotomy,^6^ induction and acceleration of labor^7^ and the Kristeller maneuver^8^ seems substantial. When indicated, these interventions are crucial in preventing maternal and perinatal morbidity and mortality; however, their routine use on healthy, low-risk women and infants can produce avoidable maternal and neonatal harm as well as increased health care costs (Seijmonsbergen-Schermers et al., 2018). Although few studies have focused on the variation over time and space in the use of these invasive procedures (Notzon, 1990; Festin et al., 2003; Tracy et al., 2007; EURO-PERISTAT Project with SCPE EUROCAT, 2013; Blondel et al., 2016), it is now certain that they vary widely even within groups of women with an identical risk profile. The instrumental delivery rate is 7.2%, with wide variations between countries: from 15.1% in Spain and Ireland to < 3% in Romania, Lithuania, Croatia, Slovakia, Slovenia, Latvia and the Czech Republic (EURO-PERISTAT Project, 2018). The figure might suggest the existence of a trade-off between instrumental and caesarean deliveries; however, a EURO-PERISTAT Project with SCPE EUROCAT (2013) analysis dated 2010 showed that countries with higher rates of instrumental deliveries do not show lower caesarean rates. Differences in intervention rates can be explained by demographic and clinical characteristics of women (such as the number of children, older maternal age, multiple births, fetal presentation and maternal obesity) but also by the peculiarities of each healthcare delivery system, as well as a number of specific factors related to it, such as fear of medical-legal litigation, financial incentives where payments are higher for CS, women's preferences for CS, and differences in clinical assessments of the risks associated with pregnancy.

Although data are old, territorial differences are also recorded looking at the use of instrumental practices during vaginal birth (Table 7).

**Table 7:** Logistical models of interventions during labor and delivery—Year 2013.

	**Episiotomy**	**Administration of oxytocin**	**Artificial rupture of membranes**	**Continuous fetal cardiac monitoring**	**Abdominal pressure**
Geographical distribution
North-west	1	1	1	1	1
North-east	0.68(0.54–0.85)	0.92 (0.72–1.17)	0.95 (0.76–1.18)	1.23(0.99–1.522)	0.94(0.72–1.22)
Center	1.05(0.83–1.33)	0.73 (0.56–0.96)	0.92 (0.72–1.17)	1.13 (0.90–1.42)	1.05(0.79–1.39)
South	1.25(1.00–1.57)	0.37(0.28–0.50)	0.65(0.52–0.82)	0.85 (0.68–1.05)	1.72(1.33–2.21)
Islands	1.53(1.18–2.05)	0.52(0.37–0.74)	1.16(0.89–1.52)	0.87 (0.66–1.14)	1.78(1.31–2.42)
Serius disorders
No	1	1	1	1	1
Yes	1.46 (1.23–1.76)	1.31(1.07–1.62)	1.43 (1.20–1.72)	1.40 (1.17–1.68)	1.46(1.19–1.78)
7 or more ultrasound scan
No	1	1	1	1	
Yes	1.22(1.04–1.44)	1.41(1.17–1.71)	1.51 (1.28–1.77)	1.29(1.10–1.51)	
**Parity**	
Multiparas	1	1			1
Primipara	1.54(1.30–1.81)	1.59(1.32–1.92)	nd	nd	2.02(1.70–2.41)
Multiple birth
No					
Yes	5.30 (1.62–17.27)	nd	3.31 (1.16–9.46)	nd	nd
Citizenship
Foreign	1	1			
Italian	1.38(1.08–1.77)	1.94(1.42–2.65)	nd	nd	nd
Age at childbearing
≤ 24	1	1			
25–29	1.09(0.79–1.50)	1.89(1.20–2.96)			
30–34	1.16(0.85–1.57)	2.39 (1.55–3.69)	nd	nd	nd
35–39	1.50(1.08–2.07)	2.60 (1.66–4.06)			
< =40	1.07(0.70–1.63)	2.16 (1.26–3.70)			

Source: ISTAT (2017).

**Figure 3:**
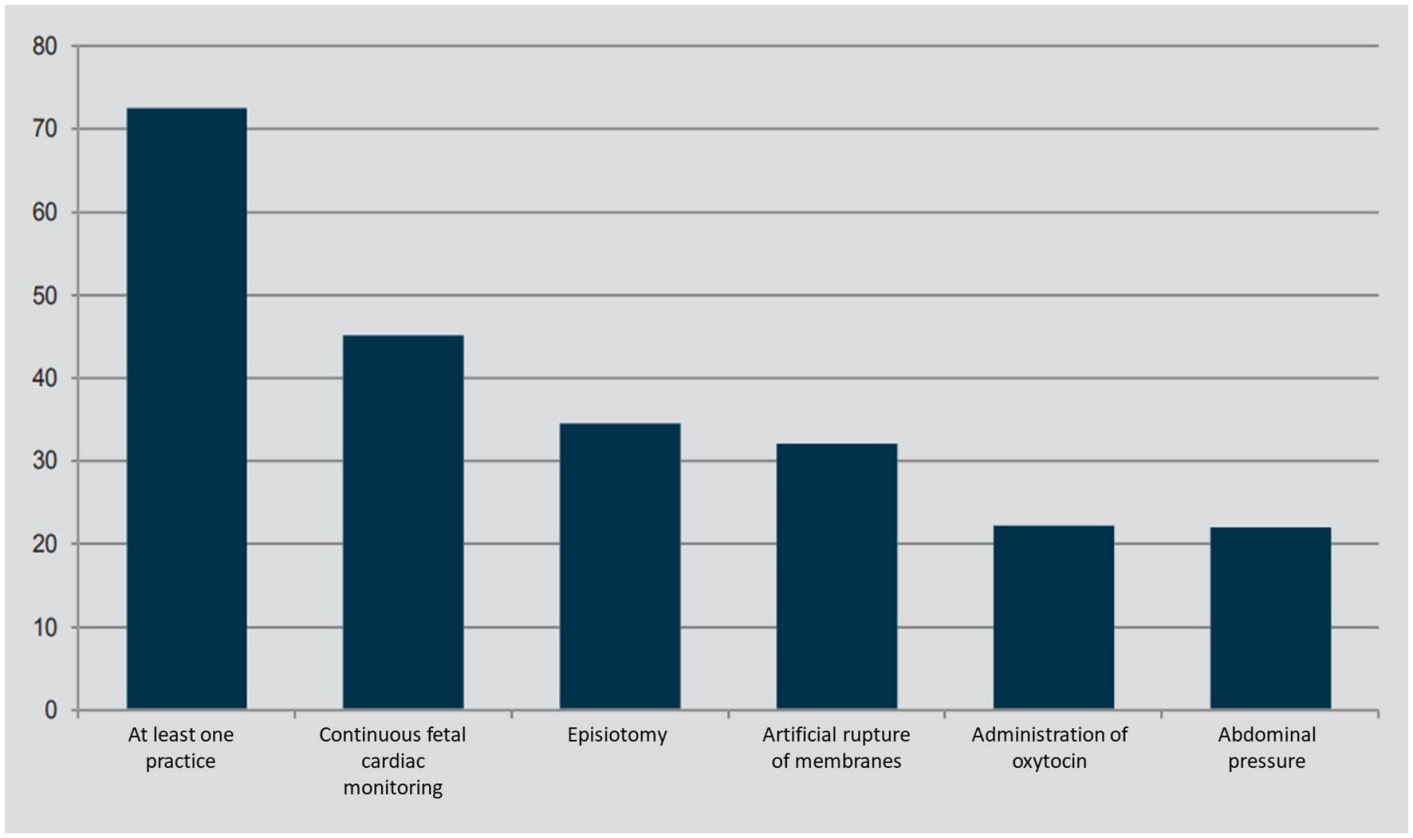
Interventions during labor and delivery—Year 2013 (per 100 women who had a spontaneous birth). Source: ISTAT (2017).

In Italy spontaneous childbirth, which affected 63.5% of women in 2020 (Ministry of Health, 2021), often turns into operative delivery.^9^ The reference here is to artificial rupture of membranes (32% of spontaneous deliveries), episiotomy (34.7%), and the administration of oxytocin to increase the frequency and intensity of contractions (22.3%). ISTAT data (2017) detected a rather high level of overall medical intervention in 2013 with 73.7% of women having undergone one of these procedures (Figure 3).

The private nature of the birthplace would not be among the factors associated with the use of such practices; instead, it would be certain characteristics of the pregnancy that put women at risk of undergoing them. The recourse to these practices may be affected by the presence of different professionals during birth. Regional differences can be observed once again: while in the central-northern regions the presence of midwives always exceeds those of gynaecologists, in the south the values tend to level off and, in some regions (Molise, Campania, Apulia and Basilicata), to invert. This could be linked to the higher incidence of surgical deliveries requiring the necessary presence of doctors.

Data highlight the centrality of the midwife, who is present in almost all childbirths (95.8%); the presence of the gynaecologist is also high and widespread (87.8%), and the presence of the paediatrician/neonatologist is lower (69.8%). The anaesthetist is present in 43.9% of cases: this percentage appears to be the result of the sum of that relating to CS and analgesia, in which this figure is required (Ministry of Health, 2021).

## 4. Discussion

The high degree of medicalization of pregnancy and birth in Italy and its strong regional variability lead one to question the existence of a national maternity model of care. Starting from the assumption that medicalization is not necessarily linked to the different health conditions of mothers in different geographical areas, one may wonder whether areas whit different sociocultural, economic, political and institutional background may have introjected different meanings of medicalization thus reproducing different maternity models of care.

What should be pointed out, in fact, is that the different meanings of medicalization of childbirth are all applicable in this analysis. Albeit they seem to trace a temporal pathway, each of them seems to still maintain its own logic, also in a changed scenario. This could be done to different context in which birth takes place. In other words, if the emergence of different meanings has a historical origin, their maintenance over time depends on the set of cultural norms prevailing in each context, on the emergence of a specific institutional configuration, on the organized action of actors involved in care and their ability to exert pressure. The possibility to apply one meaning rather than another is linked to a particular maternity model of care that prevails in each area. This, in turn, depend on the action of several contextual variables, which play an important role in shaping maternity care. After all, the same variables can explain the crystallization of a specific welfare model (Pierson, 1994; Ferrera, 1996; Kersbergen, 2000) or health system (Neri, 2006, 2008; Servetti, 2018) at national and sub-national level.

The (apparently affirmative) answer to this question seems to come from observing the simultaneous presence, in Italy, of all four different meanings of medicalization discussed above. Even with some similar traits, different conditions and situations seem to emerge in different geographical areas, leading to the prevalence of one meaning rather than another and resulting on different outcomes in terms of medicalization.

A first common trait can be seen in the constant increase in the average age of Italian motherhoods: it was passed from 31.8 years in 2004 to 33.1 years in 2021. This makes pregnancy and birth riskier, also explaining why medicalization starts in the pregnancy phase, which is often experienced as an illness.

Another common element can be seen in professional and political-institutional variable: the prevalence accorded to the medical maternity model of care stems from the strength and dominance of the medical profession. Medical power, which is particularly evident in Italy, especially in some areas of the country, can be explained looking at the professional history of this occupational group especially compared whit midwives. Their different paths of professionalization, their capacity to build alliances with political institutions and to put in place clear and effective professional strategies are very different (Spina, 2009, 2022). This asymmetry of power, that can be seen as a form of structural iatrogenesis (Illich, 1976), resulted in a different way to defend their professional jurisdictions and to obtain equal social legitimacy (Vuille, 2010). In addition, the gender connotation of the professions (midwifery, almost entirely composed by women, and gynaecology, predominantly male, at least until the recent past) has also weighed on their power. Thus, gender dynamics attributable to patriarchy have also been triggered, fuelling logics of dominance and subordination of one profession over the other (Benoit et al., 2005, 2010; Spina, 2009). This has led, everywhere in the country, to the supremacy of the medical model of birth over the social one (Kennedy and Kodate, 2019; Spina, 2022).

However, this trait appears more evident in the south, where patriarchal logic is still more deeply rooted than in the rest of the country (Vicarelli, 1997). As a result, the first two above mentioned meanings of medicalization have been affirmed here more than elsewhere: that linked to medical control on the female bodies (Foucault, 1963) and that linked to medical imperialism, also favoured by users themselves (Conrad, 1992).

Partially different is the story in the center and north of the country, where women (and the population at large) have timidly opposed this trait, claiming their empowerment (Spina, 2022). Their more active role can be seen, for example, in the demand for alternative maternity services that has contributed to the implementation of a midwifery offer of care. Birth centers and midwifery clinics (specifically dedicated to the care of low-risk pregnancies and births) have sprung up over time. In 2017 there were 10 birth centers in Italy, all run privately, of which 9 were in the northern regions: one in Piedmont, Liguria, Emilia-Romagna, Veneto and Friuli and 4 in Lombardy.^10^

However, the emancipation of central and northern women seems not to have prevented the fourth meaning of medicalization from taking root, that linked to the concept of biomedicalization (Clarke et al., 2009). This is probably due to the better infrastructural and technological equipment in this area (Vicarelli and Spina, 2020).

More general differences are due to the north-south dualism in the development process and in resources allocation. In northern regions, maternal facilities, especially the public ones, appears to be wide enough. On the contrary the shortage and the inefficiency of the public sector in the south seem to direct users toward a private market. Here, moreover, there is a lower average percentage of women who choose to rely on the assistance of family counselling centers and midwives who attend birth preparation courses (ISTAT, 2017). Consequently, the CS sections and, more generally, the medicalization of birth is higher. In some regions, in particular, the phenomenon has assumed alarming proportions and natural childbirth is becoming an increasingly rare experience. It remains to be understood how much this inefficiency is due to the lack of dedicated investments and how much, on the contrary, the absence of the latter is caused by political incapacity to counter professional lobbies, finding in the private lobbies an obstacle to the free development of the public one.

Moreover, as a result of the reorganization process of the hospital network, 190 maternity wards have been closed in more than 10 years, leaving 419 open in 2020 (Ministry of Health, 2021). Of these, 25% (103) are small facilities, i.e. with fewer than 500 births per year. Facilities with a volume of 1,000 or more births per year account for 34.8% of the total number of births, where 62.6% of the total number of births are concentrated. The regional distributions by classes of births show regionally diversified situations. In 2020, in 6 regions (all in the center-north), more than 70% of births took place in large maternity wards (at least 1,000 births per year). The opposite is true in the southern regions where over 40% of births take place in facilities with < 1,000 annual deliveries and where medicalization tends to increase,^11^ especially CS.

According to some studies (Save the Children, 2015) the high frequency of CS rate in small centers can be explained by the low number of births and the consequent lack of experience of health personnel, who thus avoid the risk of complications from vaginal deliveries, performing surgical birth rather than the vaginal one.

CS is often performed beyond the conditions of clinical needs, due to inappropriate clinical-assistance behaviour that is rooted in some hospitals and at a territorial level (ISTAT, 2017), as well as due to the affirmation of a socio-cultural tendency that assimilates it to a choice in the mode of delivery, demonstrating how users contribute to the medicalization of birth. Sometimes, the choice to perform a CS seems to be due to a way of simplifying procedures linked to structural and organizational shortcomings of maternity wards, to the fear of medical-legal litigation that “together with a progressive reduction in the competence of health personnel in managing the physiology of pregnancy and childbirth, promotes CS as a defensive practice” (Save the Children, 2015, p. 34).

## 5. Conclusions

The aim of the article was both to highlight the Italian peculiarity in terms of high medicalization of childbirth (compared to European countries), and its territorial variability. An attempt was made to see if the different meanings of medicalization were present in the different geographic areas because of their different characteristic in economic, political, institutional and professional terms. A different prevalence of these meanings in the central-north and south seems to emerge, thus resulting in different outcomes in terms of medicalization. This dualism is also visible in several attempts that was made to de-medicalise birth. In Italy, some attempts have been made in this direction, by adopting measures to humanise it such as, for example, the recommendation to allow the woman the freedom to assume the desired positions during labor and delivery, the possibility of allowing a person whom the mother trusts to enter the delivery room, and the encouragement of breast-feeding by favouring skin-to-skin contact between mother and child and allowing rooming-in. These actions, however, were implemented unevenly across the country once again. ISTAT classified the Regions into three categories: the virtuous ones, mainly located in the center-north (Province of Bolzano, Valle d'Aosta, Trentino Alto Adige Region, Province of Trento, Emilia-Romagna, Marche, Friuli, Tuscany and Piedmont), those that are neither particularly virtuous nor averse to good practices, which include several regions along the peninsula (Veneto, Lombardy, Liguria, Sardinia, Umbria, Basilicata, Apulia) and finally the regions, mainly in the south, where the spread of such practices is completely insufficient (Lazio, Abruzzo, Molise, Campania, Calabria, Sicily) (Save the Children, 2015). De-medicalising birth remains a very difficult goal.

The data presented in this article seem to deny the existence of a national maternity model of care and. On the contrary, they confirm the idea that medicalization is not necessarily linked to the different health conditions of mothers in different geographical areas and that a path dependent variable is able to explain it.

## 6. Contribution and limitations of this study

Highlighting geographical differences in medicalization of childbirth, the paper shows the need for a deeper investigation at local level. It suggests a critical interpretation of studies based on national comparison that, neglecting local contexts, risk giving an inexact representation of social reality.

However, the absence of some comparable data at both international and inter-regional level limits the analysis and the search for correlations with variables useful to explain the differences among geographical areas. Also completely missing is a focus on immigrant women for whom many considerations are not applicable.

## Author contributions

The author confirms being the sole contributor of this work and has approved it for publication.

## Footnotes

^1^Serious disorders include threatened miscarriage, threatened preterm delivery, diabetes, preeclampsia and hypertension.

^2^Until 2019, the LEA Grid was the tool with which the regions' fulfilment of the Essential Levels of Care provided by the Ministry of Health was certified. From January 2020, the New Guarantee System (NSG) for monitoring health care came into force, replacing the LEA Grid.

^3^In Italy it is possible to carry out intramural freelance work, also called “intramoenia”. It allows hospital doctors to provide services outside normal working hours, using the hospital's own outpatient and diagnostic facilities against payment of a fee by the patient.

^4^Family counselling centers are integrated primary health and social services with multidisciplinary competences, crucial for promotion and prevention in the areas of women's health, developmental age, adolescence and couple and family relationships.

^5^Episiotomy is defined as the surgical incision of the perineum made in the latter part of the second stage of labor, performed to widen the birth canal and facilitate expulsion of the cephalic extremity, reducing spontaneous vagino-perineal tears (Thacker and Banta, 1983).

^6^Artificial rupture of membranes is the artificial rupture of the sac containing the amniotic fluid, regardless of the time (before or during labor) or indication (e.g., for induction of labor).

^7^Induction of labor is a technique for artificially stimulating uterine contractions before they occur spontaneously; it is usually done by administering oxytocin,

^8^It consists of applying manual pressure to the uterine fundus during contraction and maternal pushing in the direction of the birth canal with the aim of reducing the duration of the second stage of labor.

^9^Spontaneous or “eutocic” birth is that which occurs naturally and without external intervention and differs from 'dystocial' birth, which occurs when complications occur. Childbirth is defined as 'operative' when it requires an instrumental or manual intervention that can be performed by an operator by vaginal route or by caesarean section.

^10^24 Data available at: www.nascereacasa.it.

^11^Negligible are home births estimated at 0.07% of total births in 2020.
